# Phylogenetic Insights Reveal New Taxa in Thyridariaceae and Massarinaceae

**DOI:** 10.3390/jof10080542

**Published:** 2024-08-02

**Authors:** Wen-Hui Tian, Yan Jin, Yue-Chi Liao, Turki KH. Faraj, Xin-Yong Guo, Sajeewa S. N. Maharachchikumbura

**Affiliations:** 1 Center for Informational Biology, College of Life Science and Technology, University of Electronic Science and Technology of China, Chengdu 611731, China; wenhuitian@std.uestc.edu.cn (W.-H.T.); jinyan4759@163.com (Y.J.); yuechicoco@163.com (Y.-C.L.); 2 Department of Soil Science, College of Food and Agriculture Sciences, King Saud University, P.O. Box 145111, Riyadh 11362, Saudi Arabia; talasiri@ksu.edu.sa; 3 College of Life Science, Shihezi University, Shihezi 832000, China

**Keywords:** Dothideomycetes, *Helminthosporium*, multi-locus, phylogeny, Sichuan Province, taxonomy

## Abstract

Pleosporales is a highly diverse (and the largest) order in Dothideomycetes, and it is widespread in decaying plants in various environments around the world. During a survey of fungal diversity in Sichuan Province, China, specimens of hyphomycetous and *Thyridaria*-like fungi were collected from dead branches of pine trees and cherry trees. These taxa were initially identified as members of Massarinaceae and Thyridariaceae through morphological examination. Phylogenetic analyses of the Thyridariaceae, combining ITS, SSU, LSU, *RPB2*, and *TEF1* sequence data, indicated a distinct clade sister to *Pseudothyridariella* and *Thyridariella*, distinct from any genus in the family. Thus, a new genus, *Vaginospora*, is proposed to accommodate the type species *Vaginospora sichuanensis*, which is characterized by semi-immersed globose to oblong ascomata with an ostiolar neck, cylindrical to clavate asci with an ocular chamber, and hyaline to dark brown, fusiform, 3–5-transversely septate ascospores with an inconspicuous mucilaginous sheath. Based on the morphological comparisons and multi-locus phylogenetic analyses (ITS, SSU, LSU, *RPB2*, and *TEF1*) of the Massarinaceae, we have identified three collections belonging to the genus *Helminthosporium*, leading us to propose *H. filamentosa* sp. nov., *H. pini* sp. nov., and *H. velutinum* as a new host record. According to Phylogenetic analysis, *H. pini* formed an independent clade sister to *H. austriacum* and *H. yunnanense*, and *H. filamentosa* represents the closest sister clade to *H. quercinum*. *Helminthosporium pini* is distinct from *H. austriacum* by the shorter conidiophores and *H. yunnanense* by the longer and wider conidia. The *H. filamentosa* differs from *H. quercinum* in having longer conidiophores and smaller conidia. This study extends our understanding of diversity within Thyridariaceae and *Helminthosporium*. Our findings underscore the rich biodiversity and potential for discovering novel fungal taxa within these groups.

## 1. Introduction

Pleosporales is the largest order in the Dothideomycetes and is primarily characterized by flask-shaped pseudothecia [1,2]. The diversity of species in Pleosporales is high, and most species are saprobes on decaying plant material in freshwater, marine, or terrestrial environments [3,4,5,6]. The members of Pleosporales can also be epiphytes, endophytes, or parasites on living leaves or stems, as well as hyperparasites on fungi or insects [7,8,9].

The family Thyridariaceae was proposed by Hyde et al. [10] to accommodate the genus *Thyridaria* Sacc. There are eight genera accommodated in Thyridariaceae: *Chromolaenomyces* Mapook & K.D. Hyde, *Cycasicola* Wanas., E.B.G. Jones & K.D. Hyde, *Liua* Phookamsak & K.D. Hyde, *Parathyridaria* Jaklitsch & Voglmayr, *Parathyridariella* Prigione, A. Poli, E. Bovio & Varese, *Pseudothyridariella* Mapook & K.D. Hyde, *Thyridaria* Sacc., and *Thyridariella* Devadatha, V.V. Sarma, K.D. Hyde, Wanas. & E.B.G. Jones [1,11]. The members of this family are mostly sexually morphic, characterized by having immersed or semi-immersed, globose, coriaceous, and black ascomata; peridium composed of two layers of *textura angularis* cells; 8-spored, bitunicate, cylindrical to subclavate, apically rounded with ocular chamber asci; ellipsoid to fusiform, brown, 2–3-septate ascospores [10]. The asexual morphs of Thyridariaceae are coelomycetous [12]. Species of this family saprobes on leaves and branches of woody plants and sometimes infect humans [12,13].

*Helminthosporium* Link was introduced by Link with *H. velutinum* Link as the type species [14]. It is an old and species-rich genus in the family Massarinaceae (Pleosporales). Several studies showed that *Helminthosporium* is polyphyletic, whose members are intermixed with taxa of *Byssothecium* Fuckel, *Haplohelminthosporium* Konta & K.D. Hyde, *Helminthosporiella* (Hern.-Restr., Sarria & Crous) Konta & K.D. Hyde, *Pseudosplanchnonema* Chethana & K.D. Hyde, and *Synhelminthosporium* Y.P. Chen & Maharachch. [15,16]. Many species of *Helminthosporium* have been identified through morphological studies, and the taxonomic history of the genus is complex and in a state of flux [17]. While many species are not congeneric with the generic type, they were reclassified into the other genera such as *Bipolaris* Shoemaker, *Curvularia* Boedijn, *Drechslera* S. Ito, and *Exserohilum* K.J. Leonard & Suggs [18,19,20]. In addition, some lignicolous species were transferred to *Ellismarsporium* R.F. Castañeda & X.G. Zhang, *Mirohelminthosporium* K. Zhang, D.W. Li & R.F. Castañeda, *Stanhughesiella* R.F. Castañeda & D.W. Li, *Varioseptispora* L. Qiu, Jian Ma, R.F. Castañeda & X.G. Zhang, and several other genera [21,22,23]. Based on phylogenetic analysis, four *Corynespora* Güssow species were transferred to the genus *Helminthosporium* [24]. *Corynespora* shares overlapping characteristics with *Helminthosporium*, such as determinate or percurrently extending conidiophores and integrated, terminal conidiogenous cells that produce distoseptate conidia, making it more difficult to distinguish the two genera based on morphology alone [25]. Most *Helminthosporium* species are established based on their asexual morph, which is characterized by porogenous, distoseptate conidia with a flat, ringed pore scar at the base, conidia that are acropleurogenously borne on septate, and erect conidiophores, which cease growth after the formation of terminal conidia [24,26,27,28].

Sichuan Province is located in a subtropical zone, where tropical and temperate flora coexist [29]. The diverse climatic environment and complex terrain provide highly favorable conditions for the development of biodiversity. Sichuan Province contains many rare plants and a huge fungal diversity that is waiting to be explored [30,31,32,33,34]. We regularly conduct fungal diversity surveys in Sichuan Province and investigate the taxonomy of fungi associated with specific plants, such as gymnosperms and cherries. During the study, a *Thyridaria*-like fungus and four hyphomycetous fungi were collected from *Pinus* spp. and *Prunus* spp. Based on morphological characteristics and multi-locus phylogenetic analysis, we identified these four collections as a new genus, *Vaginospora*, in the Thyridariaceae, along with two new species and a new host record of *Helminthosporium*.

## 2. Materials and Methods

### 2.1. Sample Collection, Morphological Examination, and Isolation

We surveyed the fungal diversity of Ascomycota on gymnosperms and cherries in Sichuan Province, China, from April 2023 to April 2024. The specimens were taken into the laboratory in paper envelopes for examination. Microscopic characters were observed and recorded using a Nikon SMZ800N stereo microscope equipped with a Nikon DS-Fi3 camera (Nikon Corporation, Tokyo, Japan) and a Nikon ECLIPSE Ni-U microscope (Nikon Corporation, Tokyo, Japan) fitted with a Nikon DS-Ri2 microscope (Nikon Corporation, Tokyo, Japan) camera. Measurements were conducted using the Nikon NIS-Elements Documentation Imaging Version 5.21.00 (Nikon Corporation, Tokyo, Japan). All photographs were processed using Adobe Photoshop version 22.0 (Adobe Inc., San Jose, SA, USA). Single ascospore or conidium isolation was performed following the method described by Senanayake et al. [35]. Germinated ascospore or conidia were individually transferred to potato dextrose agar (PDA) media plates and incubated in the dark at 25 °C. Culture characteristics were examined and recorded regularly after 1–3 weeks.

The holotype specimens were deposited in the Herbarium of Cryptogams Kunming Institute of Botany Academia Sinica (HKAS), Kunming, China, and all specimens were deposited in the Herbarium of the University of Electronic Science and Technology (HUEST), Chengdu, China. The living ex-type cultures were deposited in the China General Microbiological Culture Collection Center (CGMCC) in Beijing, China, and all living cultures were deposited in the University of Electronic Science and Technology Culture Collection (UESTCC), Chengdu, China. The taxonomic descriptions of the new taxa have been deposited in MycoBank.

### 2.2. DNA Extraction, PCR Amplification, and Sequencing

Fungal genomic DNA was extracted from mycelia using the Trelief^TM^ Plant Genomic DNA Kit (TSINGKE Biotech, Shanghai, China) according to the manufacturer’s protocol. For *H. pini* specimens, obtaining a culture was not feasible, necessitating the direct extraction of DNA from fruiting structures using the method used by Wanasinghe et al. [36]. Five loci—the nuclear ribosomal internal transcribed spacer (ITS: ITS1-5.8S-ITS2), the nuclear ribosomal small subunit rRNA (SSU), the nuclear ribosomal large subunit rRNA (LSU), the partial translation elongation factor 1-alpha (*TEF1*), and the partial second largest subunit of RNA polymerase II (*RPB2*)—were amplified by polymerase chain reaction (PCR). The primers used were ITS9mun/ITS4_KYO1 [37,38] for ITS, LR0R/LR5 [39,40] for LSU, PNS1/NS41 [41] for SSU, EF1-728F/EF1-2218R [42,43] for *TEF1*, and fRPB2-5F/fRPB2-7cR [44,45] for *RPB2*. The final reaction volume of the PCR reagent was 25 µL, containing 2 µL of the DNA template, 1 µL each of the forward and reverse primers, 8.5 µL of double-distilled water (ddH_2_O), and 12.5 µL of 2× Flash PCR MasterMix (mixture of DNA Polymerase, dNTPs, Mg^2+^, and optimized buffer; CoWin Biosciences, Nanjing, China). The amplification conditions for all five loci consisted of initial denaturation at 94 °C for 3 min, followed by 35 cycles of 30 s at 94 °C, 30 s at 56 °C, and 1 min at 72 °C, and a final extension period of 10 min at 72 °C. The PCR products were visualized by 1% agarose gel electrophoresis. Sanger sequencing was conducted by Tsingke Biological Technology (Beijing, China). Newly generated sequences were deposited in GenBank, and the accession numbers are listed in Table 1 and Table 2.

### 2.3. Phylogenetic Analyses

According to the corresponding Sanger sequencing chromatograms, misleading data from the ends of raw sequencing fragments were manually trimmed and assembled into consensus sequences using SeqMan Pro version 7.1.0 (DNASTAR, Inc., Madison, WI, USA). Barcode sequences of all species (Table 1 and Table 2) were downloaded from the NCBI nucleotide database using the R package Analysis of Phylogenetics and Evolution 5.0 (APE, http://ape-package.ird.fr, 26 June 2024) [46].

The multiple sequence alignments were conducted using MAFFT version 7.310 [47] with options “--adjustdirection --auto,” and the alignment files were further trimmed using trimAl version 1.4 [48] with the option “-gapthreshold 0.5”, which only allows 50% of taxa with a gap in each site. The best-fit nucleotide substitution models for each locus were selected using ModelFinder version 2.1.1 [49] under the Corrected Akaike Information Criterion (AICC). All sequence alignments were combined using an in-house Python script.

Maximum Likelihood (ML) and Bayesian analysis (BI) were conducted based on individual and combined datasets. Two phylogenetic trees were constructed by combining the ITS, SSU, LSU, *RPB2*, and *TEF1* gene regions. The first tree represents the phylogenetic analysis of the Massarinaceae, while the second tree represents the phylogenetic analysis of the Thyridariaceae. ML phylogenetic trees were obtained using the IQ-TREE version 2.0.3 [50], and the topology was evaluated using 1000 ultrafast bootstrap replicates. The BI was conducted using parallel MrBayes version 3.2.7a [51]. The ML trees were visualized using ggtree version 2.4.1 [52] and further edited in Adobe Illustrator version 16.0.0.

## 3. Results

### 3.1. Phylogenetic Analyses

**Figure 1:**
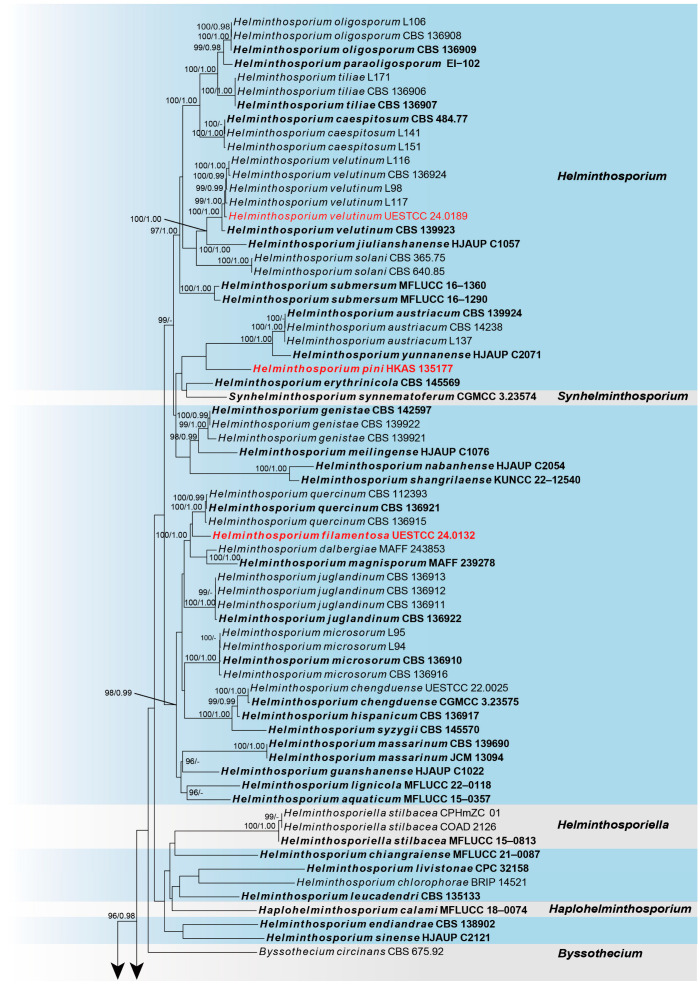

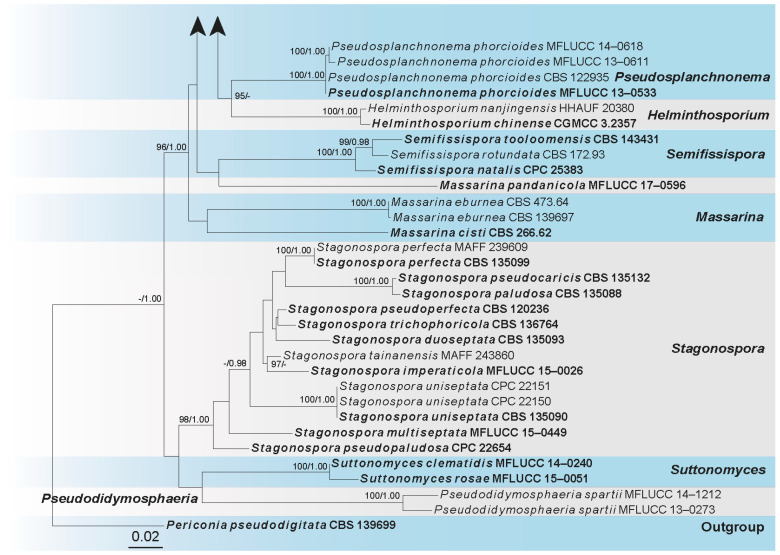
The phylogram of the family Massarinaceae from ML analysis is based on the concatenated dataset of ITS-LSU-SSU-*RPB2*-*TEF1*. The tree is rooted with *Periconia pseudodigitata* (CBS 139699). Support values of ML-UFBoot ≥ 95 and Bayesian posterior probabilities ≥ 0.95 were displayed at the nodes as ML/PP. Support values below 95 and 0.95 are indicated by a hyphen (-). Newly collected taxa are shown in red. Strains from type materials are in bold.

Sequences of Five loci were successfully obtained for *Helminthosporium filamentosa* (UESTCC 24.0132), *H. pini* (HKAS 135177), and *H. velutinum* (UESTCC 24.0189). A phylogenetic tree of all *Helminthosporium* species and representative strains from other genera in Massarinaceae was constructed (Figure 1), including 100 taxa, with *Periconia pseudodigitata* (CBS 139699) as the outgroup. The combined dataset (ITS: 1–564, LSU: 565–1415, SSU: 1416–2440, *RPB2*: 2441–3555, *TEF1*: 3556–4781) was composed of 1872 distinct patterns, 1183 parsimony-informative sites, 340 singleton sites, and 3257 constant sites. The best-fit evolution models were GTR+F+I+G4 for the ITS partitions, GTR+F+I+G4 for the LSU partition, K2P+G4 for the SSU partition, HKY+F+I+G4 for the *RPB2* partition, and GTR+F+I+G4 for the *TEF1* partition. The best-scoring ML tree (lnL = −32012.540) with support values from ML and Bayesian analysis at the node is shown in Figure 1.

According to the multi-locus phylogeny (Figure 1), our collection (HKAS 135177) formed an independent clade sister to *H. yunnanense* (HJAUP C2071) and strains of *H. austriacum*. Our collection (UESTCC 24.0132) formed a separate branch, sister to *H. dalbergiae* (MAFF 243853), *H. magnisporum* (MAFF 239278), and strains of *H. quercinum*. Our collection (UESTCC 24.0189) nests with *H. velutinum* strains with 99% ML, 1.00 PP statistical support. Combining the morphological evidence with phylogeny, we propose two new species, *H. filamentosa* (UESTCC 24.0132) and *H. pini* (HKAS 135177), isolated from *Pinus* sp. and *Prunus pseudocerasus*, respectively. Additionally, we report our collections (UESTCC 24.0189) as a new host record of *H. velutinum* from *Pinus* sp.

**Figure 2:**
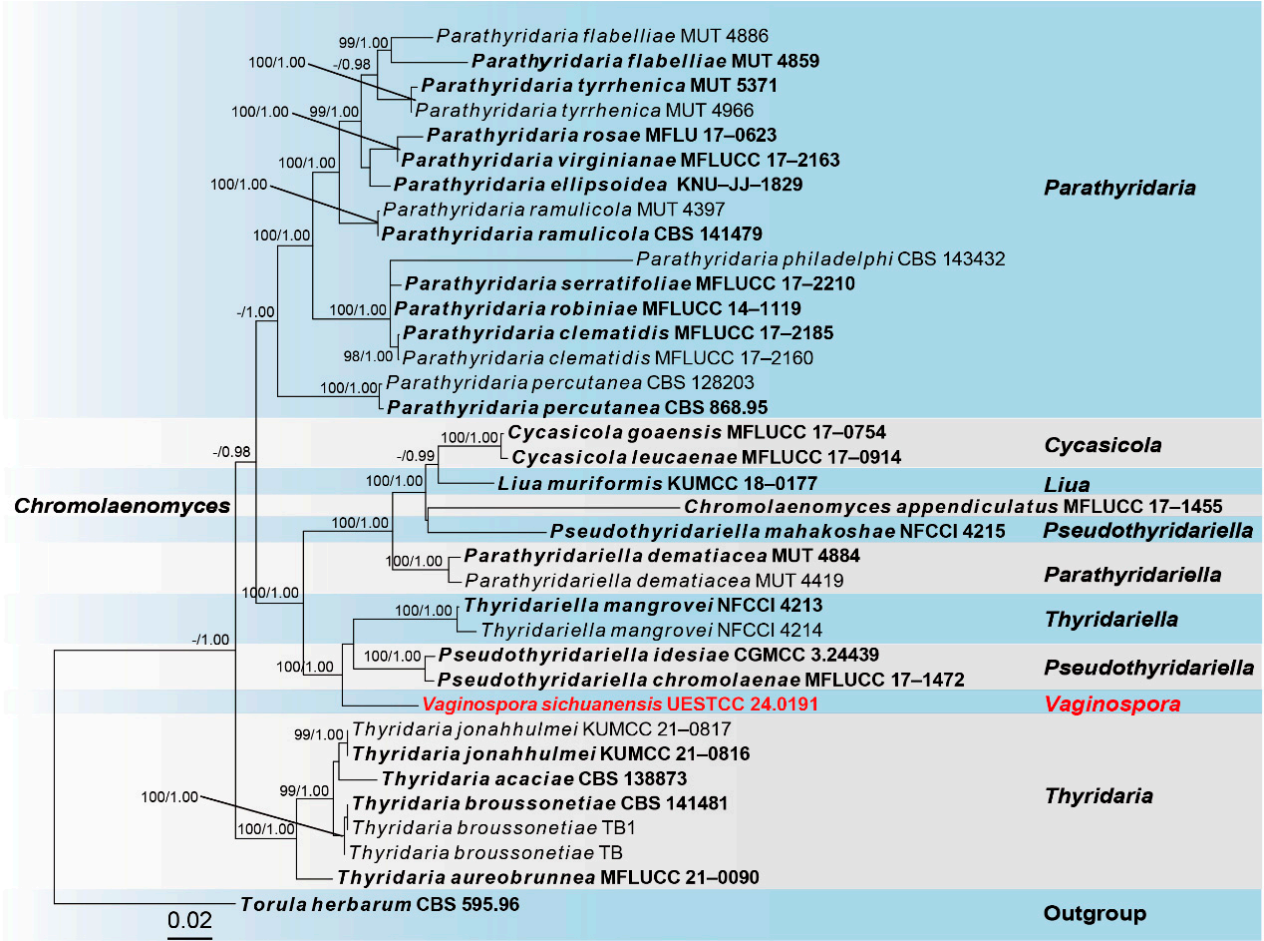
The phylogram of the family Thyridariaceae from ML analysis is based on the concatenated dataset of ITS-LSU-*RPB2*-SSU-*TEF1*. The tree is rooted with *Torula herbarum* (CBS 595.96). Support values of ML-UFBoot ≥ 95 and Bayesian posterior probabilities ≥ 0.95 were displayed at the nodes as ML/PP. Support values below 95 and 0.95 are indicated by a hyphen (-). Newly collected taxa are shown in red. Strains from type materials are in bold.

Sequences of Five loci were successfully obtained for the *Vaginospora sichuanensis* (UESTCC 24.0191). A phylogenetic tree of representative strains from other genera in Thyridariaceae was constructed (Figure 2), including 36 taxa, with *Torula herbarum* (CBS 595.96) as the outgroup. The combined dataset (ITS: 1–516, LSU: 517–1372, *RPB2*: 1373–2402, SSU: 2403–3422, *TEF1*: 3423–4307) was composed of 1448 distinct patterns, 824 parsimony-informative sites, 518 singleton sites, and 2965 constant sites. The best-fit evolution models were SYM+G4 for the ITS partitions, SYM+I for the LSU partition, SYM+I+G4 for the *RPB2* partition, K2P+G4 for the SSU partition, and GTR+F+I+G4 for the *TEF1* partition. The best-scoring ML tree (lnL = − 21563.517) with support values from ML and Bayesian analysis at the node is shown in Figure 2.

According to the multi-locus phylogeny (Figure 1), our collection (UESTCC 24.0191) formed an independent clade sister to *Pseudothyridariella chromolaenae* (MFLUCC 17–1472), *Pseudothyridariella idesiae* (CGMCC 3.24439), and strains of *Thyridariella mangrovei*. Based on the morphological evidence and phylogeny, we propose a new genus, *Vaginospora*, with the type species *Vaginospora sichuanensis*.

### 3.2. Taxonomy

**Figure 3:**
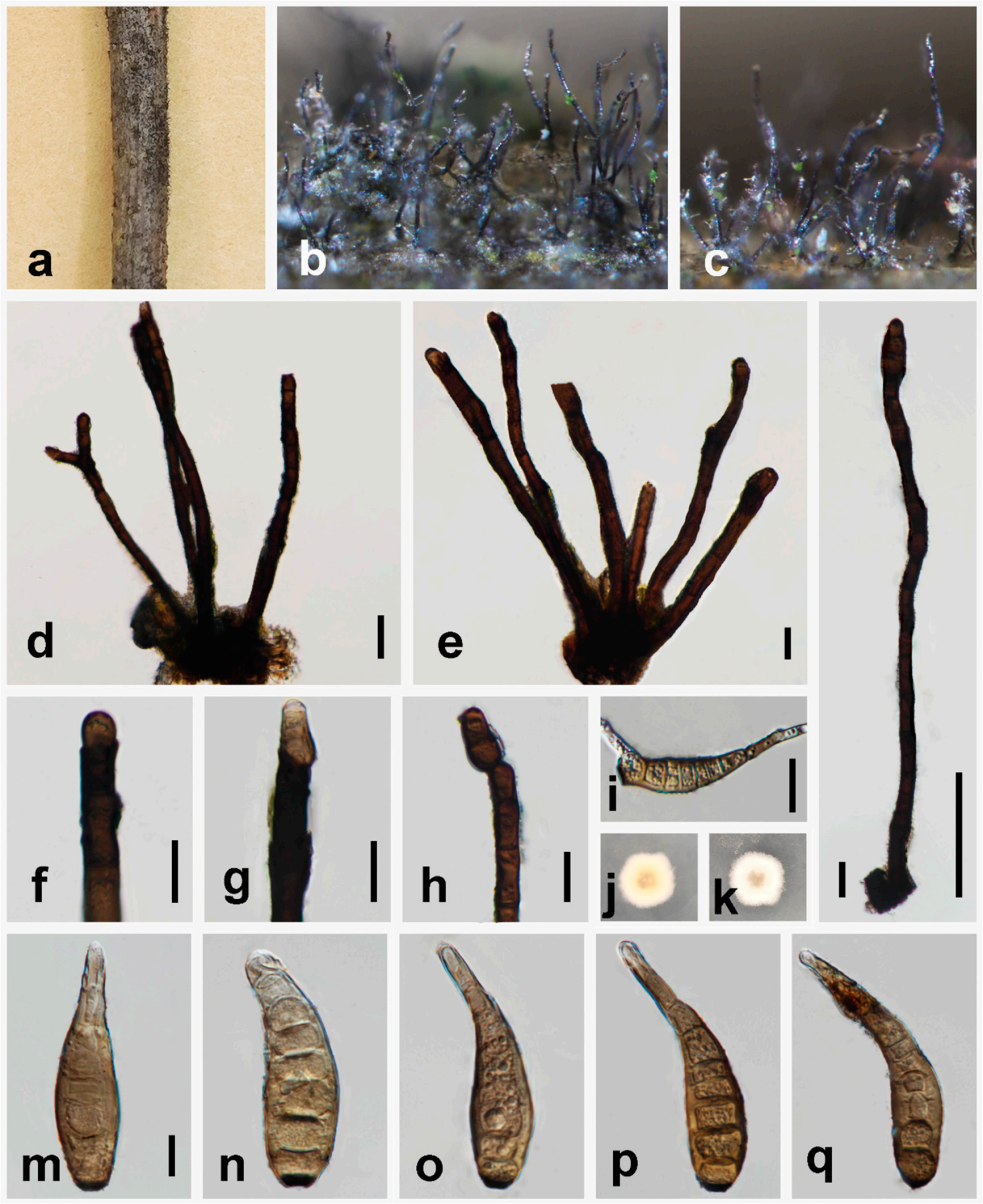
*Helminthosporium filamentosa* (HKAS 135176, holotype). (**a**–**c**) Colonies on the natural substrate; (**d**,**e**) colony and conidiophores; (**f**–**h**) conidiophores, conidiogenous cells and apical conidia; (**i**) germinating conidium; (**j**,**k**) culture characteristics on PDA after 10 days (forth and back); (**l**) solitary conidiophores and apical conidia; (**m**–**q**) conidia. Scale bars: 20 μm (**d**–**i**); 100 μm (**l**); 10 μm (**m**–**q**); scale bar (**m**) applies to (**m**–**q**).

***Helminthosporium filamentosa*** W.H. Tian, Y. Jin & Maharachch., sp. nov. (Figure 3).

*MycoBank*: MB 854338

*Etymology*: The epithet refers to the filamentous mycelium, from the Latin “*filamentum*” for filaments.

*Saprobic* on dead branch of *Prunus pseudocerasus*. in terrestrial habitats. **Asexual morph:** Hyphomycetes. *Colonies* on the natural substratum effuse, scattered, hairy, black. *Mycelium* superficial, numerous, hairy, black, scattered, solitary or fasciculate. *Conidiophores* 210–710 × 8–18 μm ($\bar{x}$ = 350 × 12.5 μm, n = 30), macronematous, mononematous, erect, simple or sometimes branched, straight to curved, verruculose, septate, black, cylindrical, obtuse at apex. *Conidiogenous cells* enteroblastic, integrated, terminal, cylindrical, black, verruculose. *Conidia* 43–73 × 10–16 μm ($\bar{x}$ = 60 × 13 μm, n = 30), cylindrical, obclavate, phragmoconidia, solitary, acrogenous, 7-distoseptate, obclavate, wide at the middle and lower part, straight or curved, uneven width, rounded at apex, truncate at base, brown, thick-walled, verruculose, secession schizolytic. **Sexual morph:** Undetermined.

*Material examined*: CHINA, Sichuan Province, Chengdu City, Pujiang Country, Cherry Mountain 30°16′25″ N, 103°50′9″ E, elevation 546 m, 19 October 2023, decaying branches of *Prunus pseudocerasus*, Y. Jin YTS21_3 (HKAS 135176, **holotype**), ex-type CGMCC 3.27591 = UESTCC 24.0132.

*Culture characteristics*: Colonies on PDA reaching 18.5 mm diam after 10 days at 25 °C, colonies from above: irregularly circular, white, margin entire, dense; reverse: cream at the margin, pale yellow in the center.

*Notes*: Multi-locus phylogenetic analysis indicated that our isolate (UESTCC 24.0132) constitutes an independent clade sister to *H. magnisporum* (MAFF 239278), *H. dalbergiae* (MAFF 243853), and strains of *H. quercinum* (Figure 1). Based on pairwise nucleotide comparisons, the closest hits to our isolate (UESTCC 24.0132) is *H. quercinum* (CBS 136921), and comparing the ITS, LSU, SSU, *RPB2*, and *TEF1* of their sequence revealed 96.88% (559/577 bp, gaps: 2/577 bp), 99.34% (754/759 bp, without gaps), 99.8% (1004/1006 bp, without gaps), 97.45% (763/783 bp, without gaps), and 97.44% (838/860 bp, gaps: 2/860 bp) similarity, respectively. Morphologically, our isolate (UESTCC 24.0132) differs from *H. quercinum* (CBS 136921) by color (black vs. brown to dark brown) and longer conidiophores (210–710 × 8–18 μm vs. 74–199 × 11–18 μm), and shorter and narrower conidia (43–73 × 10–16 μm vs. 78–130 × 15.3–18 μm) [24]. Among *Helminthosporium* species that lack molecular data, only *H. davillae*, *H. obpyriforme*, and *H. panici* have similar conidial sizes to our collection [15,53,54,55]. However, the conidia of our isolate (UESTCC 24.0132) are wider (10–16 μm vs. 4–6 μm) and more distosepta (7-distoseptate vs. 2–4-distoseptate) than *H. davillae* [55]. *Helminthosporium filamentosa* (UESTCC 24.0132) differs from *H. obpyriforme* in the morphology of the conidiophores (sometimes branched vs. simple; black vs. dark brown); it has relatively longer conidiophores (210–710 μm vs. 225–460 μm) and narrow conidia (10–16 μm vs. 14–19 μm) compared to *H. obpyriforme* [54]. *Helminthosporium filamentosa* (UESTCC 24.0132) has a different conidial morphology (obclavate vs. ellipsoidal-elongate) and more distoseptate conidia (7-distoseptate vs. 1–4-distoseptate) than *H. panici* [53]. Therefore, based on morphological characteristics and phylogenetic analysis results, we identified *H. filamentosa* as a novel species from *Prunus pseudocerasus* in China.

**Figure 4:**
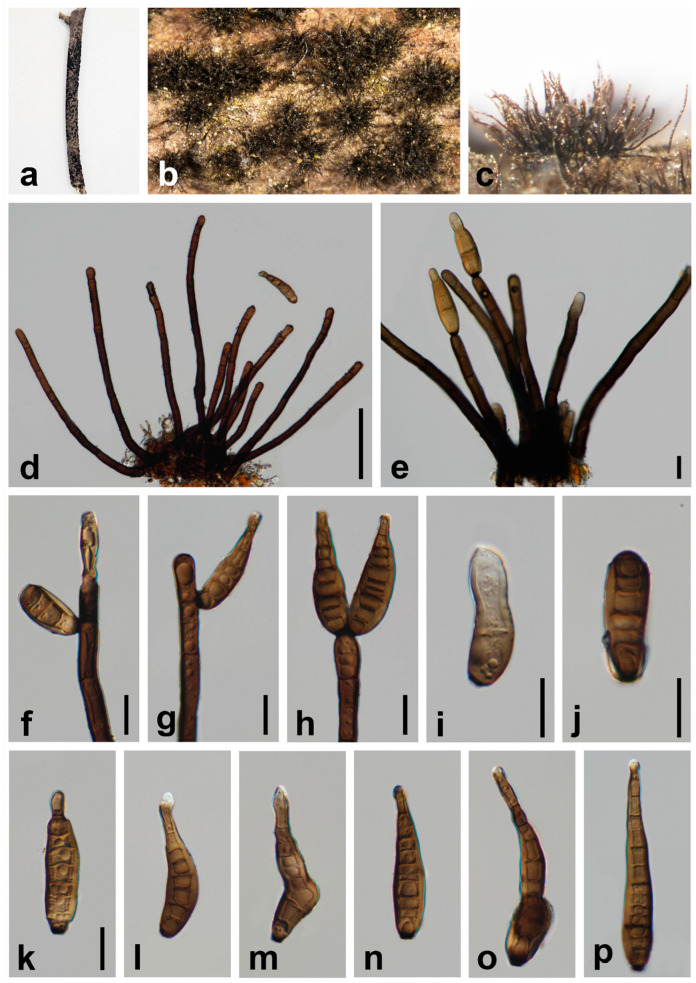
*Helminthosporium pini* (HKAS 131577, holotype). (**a**–**c**) Colonies on the natural substrate; (**d**) colony and conidiophores; (**e**) conidiophores with apical conidia; (**f**–**h**) conidiophores, conidiogenous cells, and apical conidia; (**i**,**j**) immature conidia; (**k**–**p**) conidia. Scale bars: 100 μm (**d**); 20 μm (**e**–**i**); 20 μm (**j**–**p**). Scale bar (**k**) applies to (**k**–**p**).

***Helminthosporium pini*** W.H. Tian & Maharachch., sp. nov. (Figure 4).

*MycoBank*: MB 854339

*Etymology*: Named after the host genus where the fungus was collected.

*Saprobic* on a dead branch of *Pinus* sp. in terrestrial habitats. **Asexual morph:** Hyphomycetes. *Colonies* on the natural substratum effuse, hairy, black. *Mycelium* superficial, numerous, hairy, black, scattered, solitary or fasciculate. *Conidiophores* 170–580 × 8–13 μm ($\bar{x}$ = 305 × 11 μm, n = 30), cylindrical, macronematous, mononematous, simple, erect, straight to slightly curved, verruculose, septate, dark brown to black, obtuse at apex. *Conidiogenous cells* holoblastic, integrated, terminal and intercalary, cylindrical, dark brown, verruculose. *Conidia* 60–100 × 12–18 μm ($\bar{x}$ = 80 × 16 μm, n = 30), cylindric-obclavate, phragmoconidia, solitary, acrogenous, 5–12-distoseptate, elongated, wide at the middle and lower part, straight or flexuous, uneven width, rounded at apex, obconically truncate at base, dark brown, thick-walled, verruculose, secession schizolytic. **Sexual morph:** Undetermined.

*Material examined*: CHINA, Sichuan Province, Neijiang City, Songlin Village, 29°32′19″ N, 105°9′28″ E, elevation 373 m, 1 April 2023, decaying branches of *Pinus* sp., W.H. Tian SLC11_1 (HKAS 131577, **holotype**).

*Notes*: Phylogenetic analysis based on the combined dataset of ITS, LSU, SSU, *RPB2*, and *TEF1* loci revealed that our collection (HKAS 131577) forms a separate branch sister to *H. yunnanense* (HJAUP C2071) and *H. austriacum* strains (Figure 1). In the NCBI BLASTn search, the closest matches to our isolate (UESTCC 24.0132) is *H. austriacum*. Comparing the ITS, LSU, SSU, *RPB2*, and *TEF1* of our collection (HKAS 131577) with the type strain of *H. austriacum* (CBS 139924) sequence revealed 90.87% (378/416 bp, gaps: 5/416 bp), 96.38% (718/745, gaps: 3/745 bp), 99.60% (985/989, without gaps), 90.24% (703/779, without gaps), and 94.81% (676/713, gaps: 2/713 bp) similarity, respectively. The morphological characteristics of our collection (HKAS 131577) overlap with the species of *Helminthosporium* in having septate and erect conidiophores and acro-pleurogenous and distoseptate conidia (Figure 3) [16,24]. Our collection differs from the *H. austriacum* by shorter conidiophores (170–580 μm vs. 270–920 μm [56]. Comparing the morphology of 216 *Helminthosporium* species reported worldwide, except for species with molecular sequences, only *H. gossypii*, *H. guangxiense*, *H. meliae*, and *H. pseudotsugae* had similar conidial sizes to our collection [15]. However, the conidiophores of our collection (170–580 × 8–13 μm) are longer and wider than *H. gossypii* (40–185 × 6.5–8.5 μm); shorter and narrower than *H. guangxiense* (330–850 × 8–20 μm); and longer and narrower than *H. meliae* (250–350 × 15–22 μm) [54,57,58,59]. The number of distosepta in conidia in our collection (HKAS 131577) (5–12-distoseptate) is more than *H. gossypii* (1–8-distoseptate), less than *H. guangxiense* (9–17-distoseptate), and less than *H. pseudotsugae* (8–14-distoseptate) [54,57,58,59]. Thus, our collection HKAS 131577 is described as a new species based on morphological observation and phylogenetic evidence.

**Figure 5:**
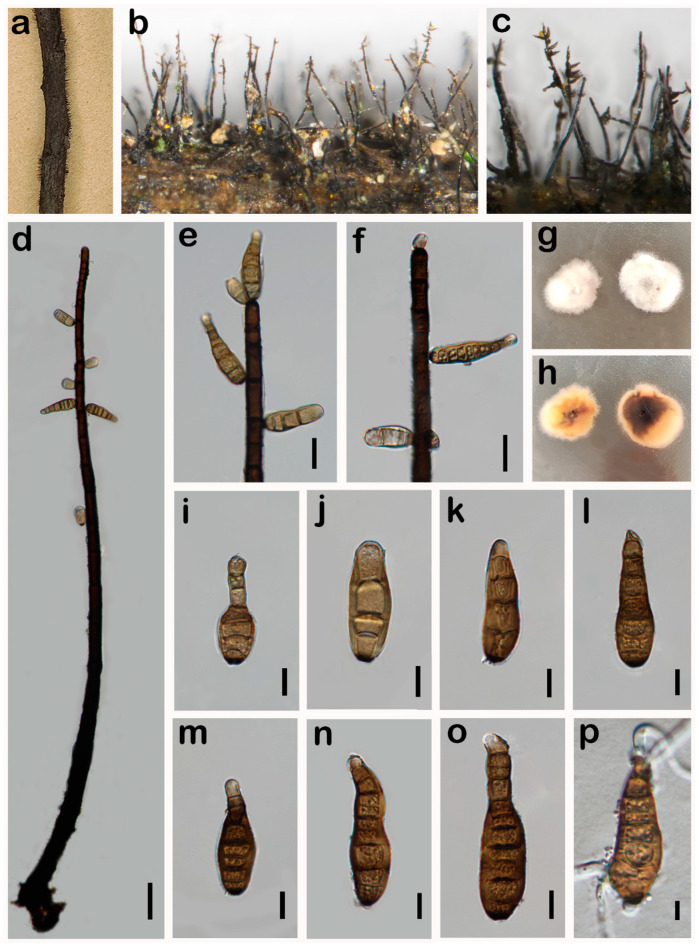
*Helminthosporium velutinum* (UESTCC 24.0189). (**a**–**c**) Colonies on the natural substrate; (**d**–**f**) conidiophores with conidia; (**g**,**h**) culture characteristics on PDA after 11 days (forth and back); (**i**–**o**) conidia; (**p**) germinating conidium. Scale bars: 100 μm (**d**); 20 μm (**e**,**f**); 10 μm (**i**–**p**).

***Helminthosporium velutinum*** W Link [as ‘Helmisporium’], Mag. Gesell. naturf. Freunde, Berlin 3(1–2): 10 (1809) (Figure 5).

*Saprobic* on a dead branch of *Pinus* sp. in terrestrial habitats. **Asexual morph:** Hyphomycetes. *Colonies* on the natural substratum effuse, hairy, black. *Mycelium* superficial, numerous, hairy, black, scattered, solitary. *Conidiophores* 275–765 × 9–12 μm ($\bar{x}$ = 525 × 16 μm, n = 30), macronematous, mononematous, unbranched, erect, straight to slightly curved, verruculose, septate, dark brown to black, cylindrical, obtuse at apex. *Conidiogenous cells* holoblastic, integrated, terminal and intercalary, cylindrical, dark brown, verruculose. *Conidia* 25.5–70 × 10–14 μm ($\bar{x}$ = 46.5 × 16 μm, n = 30), cylindric-obclavate, tapering to 4–10 μm ($\bar{x}$ = 6.5, n = 30) at the distal end, with a blackish-brown 3.5–6.5 μm wide ($\bar{x}$ = 5, n = 30) scar at the base, phragmoconidia, solitary, acrogenous, 4–10-distoseptate, wide at the middle and lower part, straight or flexuous, uneven width, rounded at apex, obconically truncate at base, brown to dark brown, thick-walled, verruculose, secession schizolytic. **Sexual morph:** Undetermined.

*Material examined*: CHINA: Sichuan Province, Chengdu City, Jiudaoguai, 30°30′21″ N, 103°53′47″ E, elevation 502 m, 19 October 2023, within dead branches of *Pinus* sp., W.H. Tian JDG31 (HUEST 24.0206), living culture UESTCC 24.0189.

*Culture characteristics*: Colony on PDA reaching 22 mm diam. in 11 days at 25 °C in the dark, colonies from above: irregularly circular, white, uneven entire, raised in center, with denser mycelium at the center; from below: yellowish brown, dark brown in the center, margin undulated.

*Notes*: *Helminthosporium velutinum* is the type species of the genus *Helminthosporium.* It is widely distributed around the world, with 110 known host records, excluding *Pinus* species [60]. *Helminthosporium velutinum* has been reported from both freshwater and terrestrial environments in China [16,24]. Multi-locus analyses of combined ITS, LSU, SSU, *RPB2*, and *TEF1* sequence data showed that our collection (UESTCC 24.0189) nests with *H. velutinum* strains with 99% ML, 1.00 PP statistical support (Figure 1). In addition, the morphological characteristics examined largely overlapped with *H. velutinum* [16]. Thus, based on morphological comparison and phylogenetic analyses, we report our collections (UESTCC 24.0189) as a new host record of *H. velutinum* from *Pinus* sp.

***Vaginospora*** W.H. Tian, Y. Jin & Maharachch., gen. nov.

*MycoBank*: MB 854941

*Etymology*: Named after its ascospores that have a sheath, from the Latin words “*vagina*” for sheath and “*spora*” for spore.

*Saprobic* on a dead branch of *Prunus* sp. in terrestrial habitats. **Sexual morph:** *Pseudothecia* solitary scattered, semi-immersed, uni-loculate, sessile, globose to oblong, ampulliform to ovoid, black, ostiolate. *Peridium* comprising several layers; outer layers dark brown to dark, with compressed cells of *textura angularis*; inner layers hyaline, with compressed pseudoparenchymatous cells, arranged in *textura angularis*. *Hamathecium* comprises dense, branched, septate, cellular pseudoparaphyses. *Asci* 8-spored, bitunicate, cylindrical to clavate, straight or curved, short pedicelate, apically rounded with an ocular chamber. *Ascospores* 2-seriate, overlapping and are initially gray-brown, turning dark brown at maturity, fusiform, mostly 3-transversely septate, sometimes 1–2 longitudinal septum appear after maturity, constricted at the septa, smooth-walled, conically rounded at the ends, verruculose, with an inconspicuous mucilaginous sheath. **Asexual morph:** undetermined.

Type species—*Vaginospora sichuanensis* W.H. Tian, Y. Jin & Maharachch.

*Notes*: According to phylogenetic analysis of Thyridariaceae, our collection (UESTCC 24.0191) constitutes a distinct clade, positioned as a sister to *Pseudothyridariella* and *Thyridariella*, with 100% ML and 1.00 PP statistical support (Figure 2). However, the sister relationship of *Vaginospora* to *Pseudothyridariella* and *Thyridariella* warrants discussion to clarify the issues of intergeneric affinities. *Thyridariella* can be distinguished from *Vaginospora* and *Pseudothyridariella* by having hyaline ascospores [61,62]. *Pseudothyridariella* produces ascospores slightly constricted at the central septum, whereas *Thyridariella* and *Vaginospora* have ascospores constricted at every septum [61,62]. Furthermore, the size of ascospores and septa in ascospores of *Vaginospora* is significantly smaller and fewer than in *Thyridariella* and *Pseudothyridariella* [61,62]. Therefore, *Vaginospora* is proposed as a new genus to accommodate *Vaginospora sichuanensis* and expand the scope of the family Thyridariaceae.

**Figure 6:**
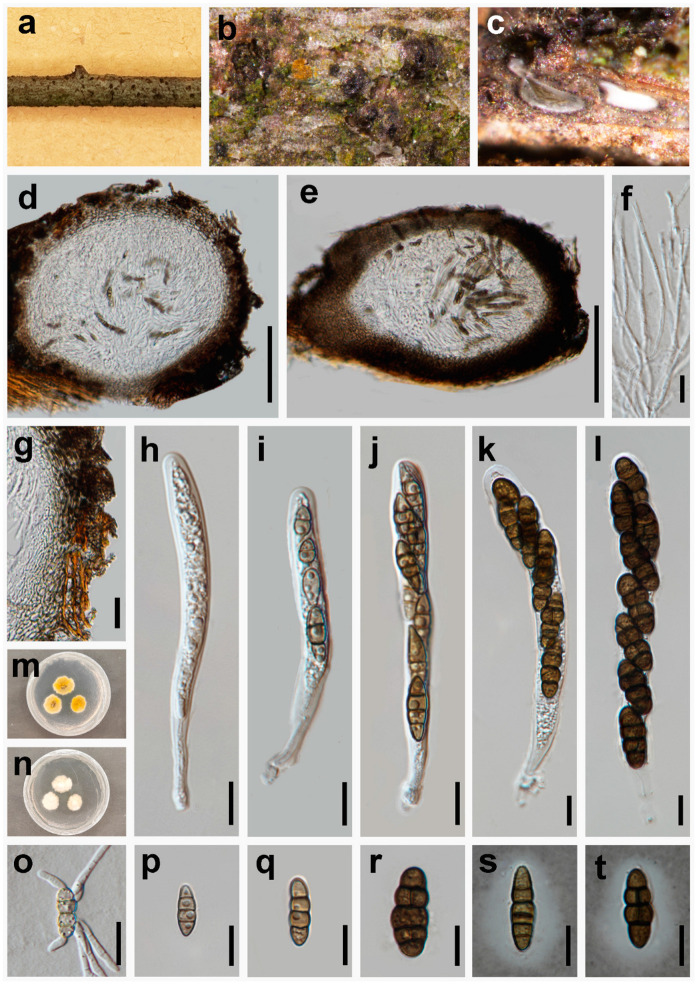
*Vaginospora sichuanensis* (UESTCC 24.0191, holotype). (**a**–**c**) Ascomata immersed in the decaying wood of *Prunus* sp.; (**d**,**e**) longitudinal sections of ascomata; (**f**) cellular and hyaline pseudoparaphyses; (**g**) section of peridium comprising cells of *textura angularis*; (**h**–**l**) immature and mature asci; (**m**,**n**) culture characteristics on PDA after 11 days (forth and back); (**o**) germinating ascospore; (**p**–**r**) ascospores; (**s**,**t**) ascospores with a mucilaginous sheath in India ink. Scale bars: 100 μm (**d**,**e**); 20 μm (**g**,**o**); 10 μm (**f**,**h**–**l**,**p**–**t**).

***Vaginospora sichuanensis*** W.H. Tian, Y. Jin & Maharachch., sp. nov. (Figure 6).

*MycoBank*: MB 854942

*Etymology*: Named after the Sichuan province, China, where the holotype was collected.

*Saprobic* on a dead branch of *Prunus* sp. in terrestrial habitats. **Sexual morph:** *Pseudothecia* 180–290 μm high × 265–375 μm diam. ($\bar{x}$ = 225 × 290 μm, n = 10), solitary scattered, semi-immersed, uni-loculate, sessile, globose to oblong, ampulliform to ovoid, black, filled by asci and ascospores and pseudoparaphyses, ostiolate. *Peridium* 28–48 μm wide, comprising several layers; outer layers dark to dark brown, with compressed cells of *textura angularis*; inner layers hyaline, with compressed pseudoparenchymatous cells, arranged in *textura angularis*. *Hamathecium* comprises dense, 1.3–2.2 μm wide, branched, septate, cellular pseudoparaphyses. *Asci* 65–140 × 6.5–15 μm ($\bar{x}$ = 90 × 10 μm, n = 20), 4–8-spored, bitunicate, cylindrical to clavate, straight or curved, short pedicelate, apically rounded with an ocular chamber. *Ascospores* 13–23 × 4–10 μm ($\bar{x}$ = 18 × 7 μm, n = 30), 2-seriate, overlapping and are initially gray-brown, turning dark brown at maturity, fusiform, muriform, mostly 3-transversely septate, sometimes 1–2 longitudinal septum appear after maturity, constricted at the septa, smooth-walled, conically rounded at the ends, verruculose, with an inconspicuous mucilaginous sheath. **Asexual morph:** undetermined.

*Material examined*: CHINA, Sichuan Province, Chengdu City, Dujiangyan County Yunhuashan, 31°00′62” N, 103°59′14” E, elevation 1535 m, 22 March 2024, decaying branches of *Prunus* sp., Y. Jin YHS46 (HKAS 136265, **holotype**), ex-type CGMCC 3.27600 = UESTCC 24.0191.

*Culture characteristics*: Colony on PDA reaching 24 mm diam. in 11 days at 25 °C in the dark, colonies from above: irregularly circular, white, uneven entire, raised in center, with denser mycelium at the center and becoming sparser at the edge; from below: yellowish brown, brown in the center, margin undulated.

*Notes*: Phylogenetic analyses of combined ITS, LSU, *RPB2*, SSU, and *TEF1* sequence data showed that our isolate (UESTCC 24.0191) forms a separate branch sister to *Pseudothyridariella* and *Thyridariella* with stable support (Figure 2). However, morphologically, our collection (UESTCC 24.0191) differs from the *P. chromolaenae* and *T. mangrovei* by narrower asci (6.5–15 μm vs. 14–22 μm vs. 10–30 μm) and smaller ascospores (13–23 × 4–10 μm vs. 23–28 × 9–12.5 μm vs. 23–40 × 8–15 μm), as well as the number of transverse septa in ascospores (1–5 septa vs. 5–8 septa vs. 5–6 septa) [61,62]. The closest matches to our isolate (UESTCC 24.0191) are species of *Pseudothyridariella* (Figure 2). Sequence comparison for the ITS, LSU, *RPB2*, SSU, and *TEF1* region between our isolate (UESTCC 24.0191) and the type species *P. chromolaenae* (MFLUCC 17–1472) showed 96.56% (365/378 bp, without gaps), 98.70% (758/768 bp, without gaps), 87.60% (636/726 bp, without gaps), 99.53% (428/430 bp, without gaps), and 95.23% (798/838 bp, without gaps) base pair similarity. Thus, our isolate UESTCC 24.0191 was identified as a new species based on morphological observation and phylogenetic evidence.

## 4. Discussion

Seven hundred and seventy-nine epithets of *Helminthosporium* have been listed in Index Fungorum (http://www.indexfungorum.org; 26 June 2024). Konta et al. compiled a list of 216 identified and accepted species of *Helminthosporium* worldwide based on species records from Index Fungorum [15]. However, most species are characterized solely based on morphology, and only 37 species have sequence data. The lack of extensive molecular data is primarily due to most species being reported and identified before sequencing technologies. This results in the possibility that many species within the *Helminthosporium* may be conspecific or belong to different genera [18,19,21,22,23]. Therefore, it is essential to re-examine the type specimens of formerly described *Helminthosporium*-like species to address this matter. Simultaneously, it is necessary to collect fresh specimens, sequence them, and combine multi-locus phylogenetic analysis with morphological examination to designate epi-types or neotypes, which is crucial for clarifying the taxonomic status of many doubtful species.

*Helminthosporium* is distributed worldwide and is mainly saprophytic on leaves or branches in terrestrial or aquatic environments [15,16,17,24,63]. Silver scurf caused by *H. solani* has become an important economic disease since the 1990s [64,65,66,67]. *Helminthosporium solani* mainly causes blemishes in the periderm of potato tubers [68]. In the 20th century, silver scurf disease also emerged in China, resulting in significant potato losses [69].

In recent studies, species of *Pseudothyridariella* have been grouped into two distinct clades based on phylogenetic analysis [70,71]. The genus *Pseudothyridariella* was proposed by Mapook et al. [62], with *P. chromolaenae* as the type species, and *Thyridariella mahakoshae* was transferred to the *Pseudothyridariella* genus as *P. mahakoshae*. However, not many sequence data and species were available for constructing phylogeny at that time. More data are emerging showing that this species is clearly unrelated to the genus *Pseudothyridariella*, which supports our study [70,71]. Morphologically, *P. mahakoshae* also differs from *P. chromolaenae* in that the former has brown or olive-brown to dark brown ascospores with 5–8 transverse septa, whereas the latter has hyaline ascospores with 3–6 transverse septate [61,62]. When the genus *Thyridariella* was introduced, it was mentioned that one of its characteristic features was the production of hyaline muriform ascospores [61]. *Pseudothyridariella idesiae* is not comparable here because it is known only for its asexual morph [71]. Our newly introduced genus, *Vaginospora sichuanensis*, has muriform ascospores that are mostly 3-transversely septate, which are morphologically and phylogenetically distinct from *P. mahakoshae*. Therefore, more evidence should be collected, and the taxonomic status of *P. mahakoshae* should be revised in future studies.

The multi-locus phylogenetic analysis in this study revealed that our newly collected taxa of *Vaginospora* represent a new genus as it constitutes a strongly supported monophyletic clade (Figure 2). Molecular evidence further indicates that *Vaginospora* belongs to the family Thyridariaceae. Our collection (UESTCC 24.0191) was morphologically similar to the type genus *Thyridaria* of Thyridariaceae, with immersed or semi-immersed ascomata, composed of *textura angularis* cells in the peridium, bitunicate, cylindrical to subclavate asci that are apically rounded with an ocular chamber, and fusiform, septate ascospores [12]. They differ in ascospore morphology, with our collection having 1–5 septa and being highly constricted at the septa, 1–2 longitudinal septa at maturity, and a sheath around the ascospores, which is not found in *Thyridaria* [12]. Morphological character comparison is a traditional classification method that cannot reflect phylogenetic relationships; it could be informative at the generic level in Thyridariaceae. For example, *Parathyridaria* was introduced into the family for its characteristic features of discoid ostiolar apices always present and pale to greyish-brown ascospores [12], *Thyridariella* is distinguished from other genera by its hyaline ascospores [61]; a typical feature of *Pseudothyridariella* is that the ascospores are constricted only at the central septum [62]. Thus, within Thyridariaceae, ascospores morphology is diverse, and when establishing a new genus, it can be combined with molecular data to provide further evidence and support.

## Figures and Tables

**Table 1:** Species details and their GenBank accession numbers used in phylogenetic analyses of Massarinaceae. Type isolates are in bold, and newly generated sequences are in red.

Species	Culture/Specimen No.	GenBank Accession Numbers				
		SSU	LSU	ITS	*RPB2*	*TEF1*
*Byssothecium circinans*	CBS 675.92	GU205235	GU205217	OM337536	DQ767646	GU349061
***Haplohelminthosporium calami***	**MFLUCC 18-0074**	**MT928160**	**MT928156**	**MT928158**	**–**	**–**
*Helminthosporiella stilbacea*	COAD 2126	**–**	**–**	MG668862	**–**	MG682500
*H. stilbacea*	CPHmZC-01	**–**	KX228355	KX228298	**–**	**–**
***H. stilbacea***	**MFLUCC 15-0813**	**MT928161**	**MT928157**	**MT928159**	**–**	**MT928151**
***Helminthosporium aquaticum***	**MFLUCC 15-0357**	**KU697310**	**KU697306**	**KU697302**	**–**	**–**
***H. austriacum***	**CBS 139924**	**KY984420**	**KY984301**	**KY984301**	**KY984365**	**KY984437**
*H. austriacum*	CBS 14238	–	KY984303	KY984303	KY984367	KY984439
*H. austriacum*	L137	–	KY984302	KY984302	KY984366	KY984438
***H. cespitosum***	**CBS 484.77**	**KY984421**	**JQ044448**	**JQ044429**	**KY984370**	**KY984440**
*H. cespitosum*	L141	–	KY984305	KY984305	KY984368	–
*H. cespitosum*	L151	–	KY984306	KY984306	KY984369	–
***H. chengduense***	**CGMCC 3.23575**	**ON557757**	**ON557745**	**ON557751**	**ON563073**	**ON600598**
*H. chengduense*	UESTC 22.0025	ON557756	ON557744	ON557750	ON563072	ON600597
***H. chiangraiense***	**MFLUCC 21-0087**	**–**	**MZ538538**	**MZ538504**	**–**	**–**
***H. chinense***	**CGMCC 3.23570**	**ON557760**	**ON557748**	**ON557754**	**–**	**ON600601**
*H. chlorophorae*	BRIP 14521	–	–	AF120259	–	–
*H. dalbergiae*	MAFF 243853	AB797231	AB807521	LC014555	–	AB808497
***H. endiandrae***	**CBS 138902**	**–**	**KP004478**	**KP004450**	**–**	**–**
***H. erythrinicola***	**CBS 145569**	**–**	**MK876432**	**NR_165563**	**MK876486**	**–**
***H. filamentosa***	**UESTCC 24.0132**	**PP835319**	**PP835316**	**PP835322**	**PP844886**	**PP844888**
***H. genistae***	**CBS 142597**	**–**	**KY984310**	**KY984310**	**KY984374**	**–**
*H. genistae*	CBS 139922	KY984423	KY984309	KY984309	KY984373	–
*H. genistae*	CBS 139921	KY984422	KY984308	KY984308	KY984372	–
***H. guanshanense***	**HJAUP C1022**	**OQ172247**	**OQ172239**	**OQ172249**	**OQ234978**	**OQ256247**
***H. hispanicum***	**CBS 136917**	**KY984424**	**KY984318**	**KY984318**	**KY984381**	**KY984441**
***H. jiulianshanense***	**HJAUP C1057**	**–**	**OQ172253**	**OQ172245**	**OQ234979**	**–**
***H. juglandinum***	**CBS 136922**	**–**	**KY984321**	**KY984321**	**KY984384**	**KY984444**
*H. juglandinum*	CBS 136911	KY984425	KY984322	KY984322	KY984385	KY984445
*H. juglandinum*	CBS 136912	–	KY984319	KY984319	KY984382	KY984442
*H. juglandinum*	CBS 136913	–	KY984320	KY984320	KY984383	KY984443
***H. leucadendri***	**CBS 135133**	**–**	**KF251654**	**KF251150**	**KF252159**	**KF253110**
***H. lignicola***	**MFLUCC 22-0118**	**OP740253**	**OP740252**	**ON329811**	**OP757656**	**OP757657**
***H. livistonae***	**CPC 32158**	**–**	**NG_064539**	**NR_160348**	**–**	**–**
***H. magnisporum***	**MAFF 239278**	**AB797232**	**AB807522**	**AB811452**	**–**	**AB808498**
***H. massarinum***	**CBS 139690**	**AB797234**	**AB807524**	**AB809629**	**–**	**AB808500**
*H. massarinum*	JCM 13094	AB797233	AB807523	AB809628	–	AB808499
***H. meilingense***	**HJAUP C1076**	**OQ172246**	**OQ172238**	**OQ172244**	**OQ234980**	**OQ234981**
***H. microsorum***	**CBS 136910**	**KY984427**	**KY984329**	**KY984329**	**KY984390**	**KY984448**
*H. microsorum*	L94	KY984426	KY984327	KY984327	KY984388	KY984446
*H. microsorum*	CBS 136916	–	KY984323	KY984323	KY984386	–
*H. microsorum*	L95	–	KY984328	KY984328	KY984389	KY984447
***H. nabanhense***	**HJAUP C2054**	**OP555400**	**OP555398**	**OP555394**	**–**	**OP961931**
*H. nanjingensis*	HHAUF020380	–	–	KF192322	–	–
***H. oligosporum***	**CBS 136909**	**–**	**KY984333**	**KY984333**	**KY984394**	**KY984451**
*H. oligosporum*	CBS 136908	KY984428	KY984332	KY984332	KY984393	KY984450
*H. oligosporum*	L106	–	KY984330	KY984330	KY984391	KY984449
***H. paraoligosporum***	**EI-102**	**–**	**OM971069**	**OM971061**	**–**	**–**
***H. pini***	**HKAS 135177**	**PP835320**	**PP835317**	**PP835323**	**PP844887**	**PP844889**
***H. quercinum***	**CBS 136921**	**KY984429**	**KY984339**	**KY984339**	**KY984400**	**KY984453**
*H. quercinum*	CBS 112393	–	KY984334	KY984334	KY984395	KY984452
*H. quercinum*	CBS 136915	–	KY984336	KY984336	KY984397	–
***H. shangrilaense***	**KUNCC22-12540**	**OP767127**	**OP767126**	**OP767128**	**–**	**OQ186449**
***H. sinense***	**HJAUP C2121**	**OP555399**	**OP555397**	**OP555393**	**–**	**OP961932**
*H. solani*	CBS 365.75	KY984430	KY984341	KY984341	KY984402	KY984455
*H. solani*	CBS 640.85	–	KY984342	KY984342	KY984403	–
***H. submersum***	**MFLUCC 16-1360**	**MG098796**	**MG098787**	**–**	**–**	**MG098586**
*H. submersum*	MFLUCC 16-1290	MG098797	MG098788	MG098780	MG098592	MG098587
***H. syzygii***	**CBS 145570**	**–**	**MK876433**	**NR_165564**	**MK876487**	**–**
***H. tiliae***	**CBS 136907**	**KY984431**	**KY984345**	**KY984345**	**KY984406**	**KY984457**
*H. tiliae*	CBS 136906	–	KY984344	KY984344	KY984405	–
*H. tiliae*	L171	–	KY984343	KY984343	KY984404	KY984456
***H. velutinum***	**CBS 139923**	**KY984432**	**KY984352**	**KY984352**	**KY984413**	**KY984463**
*H. velutinum*	CBS 136924	–	KY984347	KY984347	KY984408	KY984458
*H. velutinum*	L98	KY984433	KY984359	KY984359	KY984417	KY984466
*H. velutinum*	L116	–	KY984348	KY984348	KY984409	KY984459
*H. velutinum*	L117	–	KY984349	KY984349	KY984410	KY984460
*H. velutinum*	UESTCC 24.0189	PQ046105	PQ038268	PQ038261	PQ057762	PQ050355
***H. yunnanense***	**HJAUP C2071**	**OP555392**	**OP555396**	**OP555395**	**OP961934**	**OP961933**
***Massarina cisti***	**CBS 266.62**	**AB521718**	**AB521735**	**LC014569**	**–**	**AB808517**
*M. eburnea*	CBS 139697	GU296170	GU301840	OM337528	GU371732	GU349040
*M. eburnea*	CBS 473.64	MG646979	MG646947	MG646958	**–**	MG646986
***M. pandanicola***	**MFLUCC 17-0596**	**NG_064850**	**NG_059396**	**NR_153490**	**–**	**AB808540**
***Periconia pseudodigitata***	**CBS 139699**	**KP325438**	**KP325436**	**KP325434**	**–**	**–**
*Pseudodidymosphaeria spartii*	MFLUCC 13-0273	KP325439	KP325437	KP325435	**–**	**–**
*P. spartii*	MFLUCC 14-1212	KY984434	KY984360	KY984360	KY984418	KY984467
*P. phorcioides*	CBS 122935	KM875455	KM875454	**–**	**–**	**–**
***P. phorcioides***	**MFLUCC 13-0533**	**KP683377**	**KP683376**	**KP683375**	**–**	**–**
*P. phorcioides*	MFLUCC 13-0611	KP683374	KP683373	KP683372	**–**	**–**
*P. phorcioides*	MFLUCC 14-0618	**–**	KT950858	KT950846	**–**	KT950878
***Semifissispora natalis***	**CPC 25383**	**–**	**KT950859**	**KT950847**	**–**	**–**
*S. rotundata*	CBS 172.93	**–**	NG_058526	NR_156674	**–**	**–**
***S. tooloomensis***	**CBS 143431**	**–**	**KF251758**	**KF251255**	**KF252260**	**KF253205**
***S. duoseptata***	**CBS 135093**	**KY706138**	**KY706133**	**KY706143**	**KY706149**	**KY706146**
***S. imperaticola***	**MFLUCC 15-0026**	**–**	**NG_068239**	**NR_165854**	**–**	**–**
***S. multiseptata***	**MFLUCC 15-0449**	**–**	**KF251760**	**KF251257**	**KF252262**	**KF253207**
***S. paludosa***	**CBS 135088**	**–**	**KF251761**	**KF251258**	**KF252263**	**–**
***S. perfecta***	**CBS 135099**	**AB797289**	**AB807579**	**AB809642**	**–**	**AB808555**
*S. perfecta*	MAFF 239609	**–**	KF251763	KF251259	KF252265	KF253210
***S. pseudocaricis***	**CBS 135132**	**–**	**NG_058052**	**NR_137840**	**–**	**–**
***S. pseudopaludosa***	**CPC 22654**	**AB797287**	**AB807577**	**AB809641**	**–**	**AB808553**
***S. pseudoperfecta***	**CBS 120236**	**AB797290**	**AB807580**	**AB809643**	**–**	**AB808556**
*S. tainanensis*	MAFF 243860	**–**	NG_058081	NR_156586	KJ869232	**–**
***S. trichophoricola***	**CBS 136764**	**–**	**KF251767**	**KF251264**	**KF252269**	**–**
***S. uniseptata***	**CBS 135090**	**–**	**KF251769**	**KF251266**	**KF252271**	**KF253214**
*S. uniseptata*	CPC 22150	**–**	KF251768	KF251265	KF252270	KF253213
*S. uniseptata*	CPC 22151	KP842920	KP842917	**–**	**–**	**–**
***Suttonomyces clematidis***	**MFLUCC 14-0240**	**MG829185**	**MG829085**	**MG828973**	**–**	**–**
***S. rosae***	**MFLUCC 15-0051**	**ON557758**	**ON557746**	**ON557752**	**ON563074**	**ON600599**
***Synhelminthosporium synnematoferum***	**CGMCC 3.23574**	**ON557758**	**ON557746**	**ON557752**	**ON563074**	**ON600599**

**Table 2:** Species details and their GenBank accession numbers used in phylogenetic analyses of Thyridariaceae. Type isolates are in bold, and newly generated sequences are in red.

Species	Culture/Specimen No.	GenBank Accession Numbers				
		SSU	LSU	ITS	*RPB2*	*TEF1*
***Chromolaenomyces appendiculatus***	**MFLUCC 17–1455**	**MT214394**	**NG_068705**	**NR_168862**	**MT235806**	**MT235770**
***Cycasicola goaensis***	**MFLUCC 17–0754**	**MG829112**	**MG829001**	**MG828885**	**–**	**MG829198**
***C. leucaenae***	**MFLUCC17–0914**	**MK347833**	**MK347942**	**NR_163322**	**MK434900**	**MK360046**
***Liua muriformis***	**KUMCC 18–0177**	**MK433595**	**MK433598**	**MK433599**	**MK426799**	**MK426798**
*Parathyridaria clematidis*	MFLUCC 17–2160	MT226710	MT214599	MT310643	MT394710	MT394655
***P. clematidis***	**MFLUCC 17–2185**	**NG_070668**	**MT214598**	**MT310642**	**MT394709**	**MT394654**
***P. ellipsoidea***	**KNU–JJ–1829**	**–**	**LC552952**	**LC552950**	**–**	**–**
*P. flabelliae*	MUT 4886	KT587317	KP671720	KR014358	MN605930	MN605910
***P. flabelliae***	**MUT 4859**	**KT587315**	**KP671716**	**KR014355**	**MN605929**	**MN605909**
*P. percutanea*	CBS 128203	KF366450	KF366448	KF322117	KF366453	KF407988
***P. percutanea***	**CBS 868.95**	**KF366451**	**KF366449**	**KF322118**	**KF366452**	**KF407987**
*P. philadelphi*	CBS 143432	**–**	NG_063958	MH107905	**–**	MH108023
*P. ramulicola*	MUT 4397	MN556311	KF636775	KC339235	MN605933	MN605913
***P. ramulicola***	**CBS 141479**	**KX650514**	**KX650565**	**NR_147657**	**KX650584**	**KX650536**
***P. robiniae***	**MFLUCC 14–1119**	**–**	**KY511141**	**KY511142**	**–**	**KY549682**
***P. rosae***	**MFLU 17–0623**	**–**	**NG_059873**	**NR_157530**	**–**	**–**
***P. serratifoliae***	**MFLUCC 17–2210**	**NG_070669**	**MT214602**	**MT310646**	**MT394713**	**MT394658**
*P. tyrrhenica*	MUT 4966	KT587309	KP671740	KR014366	MN605931	MN605911
***P. tyrrhenica***	**MUT 5371**	**KU314952**	**MN556329**	**KU314951**	**MN605932**	**MN605912**
***P. virginianae***	**MFLUCC 17–2163**	**NG_070670**	**NG_073853**	**MT310647**	**MT394714**	**MT394659**
*Parathyridariella dematiacea*	MUT 4419	MN556313	KF636786	KC339245	MN605925	MN605905
***P. dematiacea***	**MUT 4884**	**KT587329**	**KP671726**	**MN556317**	**MN605926**	**MN605906**
***Pseudothyridariella chromolaenae***	**MFLUCC 17–1472**	**MT214395**	**NG_068706**	**NR_168863**	**MT235807**	**MT235771**
***P. idesiae***	**CGMCC 3.24439**	**OR253216**	**OR253307**	**OR253148**	**OR253762**	**OR251154**
***P. mahakoshae***	**NFCCI 4215**	**MG020441**	**MG020438**	**MG020435**	**MG020446**	**MG023140**
***Thyridaria acaciae***	**CBS 138873**	**–**	**KP004497**	**KP004469**	**–**	**–**
***T. aureobrunnea***	**MFLUCC 21–0090**	**–**	**MZ538562**	**MZ538528**	**–**	**–**
***T. broussonetiae***	**CBS 141481**	**NG_063067**	**–**	**NR_147658**	**–**	**–**
*T. broussonetiae*	TB	**–**	**–**	KX650567	KX650585	KX650538
*T. broussonetiae*	TB1	KX650515	**–**	KX650568	KX650586	KX650539
***T. jonahhulmei***	**KUMCC 21–0816**	**ON007046**	**ON007037**	**ON007041**	**ON009135**	**ON009131**
*T. jonahhulmei*	KUMCC 21–0817	ON007047	ON007038	ON007042	ON009136	ON009132
*Thyridariella mangrovei*	NFCCI 4214	MG020442	MG020439	MG020436	MG020447	MG020444
***T. mangrovei***	**NFCCI 4213**	**MG020440**	**MG020437**	**MG020434**	**MG020445**	**MG020443**
***Torula herbarum***	**CBS 595.96**	**KF443387**	**KF443385**	**KF443408**	**KF443395**	**KF443402**
***Vaginospora sichuanensis***	**UESTCC 24.0191**	**PQ046104**	**PQ038267**	**PQ038260**	**PQ05035** **8**	**PQ050354**

## Data Availability

All sequence data are available in NCBI GenBank following the accession numbers in the manuscript.
